# Fruit Seed Biomass as an Alternative Material to Use in Recycling Processes of Metals from Industrial Waste

**DOI:** 10.3390/ma18133063

**Published:** 2025-06-27

**Authors:** Lukasz Kortyka, Jerzy Labaj, Lukasz Mycka, Tomasz Matula, Szymon Ptak, Dorota Babilas, Tomasz Wojtal, Leszek Blacha, Albert Smalcerz, Robert Findorak, Bartosz Chmiela

**Affiliations:** 1 Łukasiewicz Research Network—Institute of Non-Ferrous Metals, Sowinskiego Street 5, 44-100 Gliwice, Poland; lukasz.kortyka@imn.lukasiewicz.gov.pl (L.K.); lukasz.mycka@imn.lukasiewicz.gov.pl (L.M.); 2 Department of Production Engineering, Faculty of Materials Science, Silesian University of Technology, Krasinskiego 8, 40-019 Katowice, Poland; jerzy.labaj@polsl.pl; 3 Department of Metallurgy and Recycling, Faculty of Materials Science, Silesian University of Technology, Krasinskiego 8, 40-019 Katowice, Poland; leszek.blacha@polsl.pl; 4 Safety Engineering and Civil Protection Faculty, Fire University, 52/54 Slowackiego Street, 01-629 Warsaw, Poland; sptak@apoz.edu.pl; 5 Department of Inorganic Chemistry, Analytical Chemistry and Electrochemistry, Faculty of Chemistry, Silesian University of Technology, Ksiedza Marcina Strzody 9, 44-100 Gliwice, Poland; dorota.babilas@polsl.pl; 6 Central Mining Institute—National Research Institute, Plac Gwarków 1, 40-160 Katowice, Poland; tomaszniemam@gmail.com; 7 Department of Industrial Informatics, Faculty of Materials Science, Silesian University of Technology, Krasinskiego 8, 40-019 Katowice, Poland; albert.smalcerz@polsl.pl; 8 Institute of Metallurgy, Faculty of Materials, Metallurgy and Recycling, Technical University of Košice, Letná 1/9, 042 00 Košice, Slovakia; robert.findorak@tuke.sk; 9 Department of Materials Technology, Faculty of Materials Science, Silesian University of Technology, Krasinskiego 8, 40-019 Katowice, Poland; bartosz.chmiela@polsl.pl

**Keywords:** biomass, fruit seeds, cherry stones, metallurgical slag, pyrometallurgy

## Abstract

The metallurgical industry has been constantly changing over the past decades. On the one hand, there has been the modernization and improvement of production efficiency, and on the other hand, we have seen a reduction in the negative impact on the environment. The possibility of using alternative materials and the circular economy is significant in this area. In the present work, research was carried out to determine the usefulness of biomass in the form of fruit seeds for the recycling processes of metal-bearing raw materials, including slags from copper production processes, which are characterized by a much higher metal content than ores of this metal. The main carbon-bearing material/reducer used in the process is metallurgical coke. The transformation of the European metal industry has been observed in recent years. To carry out the physicochemical characterization of the tested material, a research methodology was adopted using tools to determine the stability of behavior at high temperatures, chemical composition, and volatile components. Thermodynamic analysis was carried out, indicating the theoretical course of reactions of individual system components and thermal effects, allowing a determination of whether the assumed reactions are endothermic or exothermic. The planned research ends with the reduction process in conditions similar to those carried out in industrial conditions. Enforced by the guidelines for reducing CO_2_ emissions, it contributes to a significant reduction in the demand for coke. This paper addresses the issue of determining the feasibility of using selected bioreducers, including cherry stones, to verify their suitability in the process of reducing copper oxides. The study used copper slag with a composition similar to slags generated at the copper production stage in a flash furnace. The results obtained in reducing copper content above 98 wt. % indicate the great potential of this type of bioreducer. It should be noted that, unlike conventional fossil fuels, the use of cherry stones to reduce copper slag can be considered an environmentally neutral method of carbon offset. The resulting secondary slag is a waste product that can be stored and disposed of without harmful environmental effects due to its low lead content. An additional advantage is the relatively wide availability of cherry stones.

## 1. Introduction

The climate changes observed over the past decades have led to a search for the causes and effects of these changes. A significant increase in the atmosphere’s carbon dioxide content is indicated among the various factors. As a result, legal solutions have been proposed to curb the anthropogenic increase in carbon dioxide emissions, leading to zero emissions, which is defined as the balance between the adsorbed and emitted amounts of carbon compounds in production processes. These processes mainly include power generation from the combustion of coal and fossil fuels and reduction processes for obtaining metals.

One of the basic raw materials used in metallurgical processes is coke, mainly directed at pyrometallurgical technologies to obtain metals, or foundry and heating processes. Currently, the metallurgical industry consumes about 75% of the total amount of coke produced, with an annual production of coke of 702 million tonnes, 80% of which is produced in Asian countries [1]. The transformation of the European steel industry observed in recent years, mainly enforced by the guidelines for reducing CO_2_ emissions, has contributed to a significant reduction in the demand for coke. It can be assumed that the displacement of shaft furnace smelting technologies (including the blast furnace process) by technologies based on the use of natural gas and, in the long term, hydrogen as an energy carrier, as well as the replacement of coke with various types of biomass, will result in a significant drop in demand for this raw material [2,3].

The current article discusses the possibility of using widely available biomass, such as cherry stones, to recover metals from industrial waste generated in the copper production process. Coke or coke breeze enables the metal oxide reduction reaction in this process. Its consumption depends mainly on the content of the metals we want to recover and the technology used. Considering the two-stage technology for obtaining copper using a shaft furnace and a Peirce–Smith-type converter, the copper content in the slag does not exceed the level of 0.5 wt. % after the shaft process, and in the slags obtained after the converter stage, the copper content is in the range of 6–8 wt. %. A much higher copper content characterizes the slags obtained when processing the concentrate in a single-addition slurry furnace technology (11–18 wt. %). Due to their high copper content, secondary raw materials are processed in electric furnaces, with an average coke consumption of 40–60 kg/ton of slag [4].

Up to 4% copper in the copper concentrate is assumed to be lost in waste slag. Thus, any measures aimed at reducing these losses, e.g., by improving the efficiency of copper recovery from them, are considered expedient. An economic prerequisite for using secondary raw materials in a closed-loop economy, including the recovery of metals from slag, is the cost of such a process, which must not exceed the value of the recovered metals. The economic balance sheet must also consider indirect costs, including environmental costs incurred by the company, resulting not only from the price of disposing of waste slag (if it is not managed) but also from ETS (Emissions Trading System) costs.

Investigating the suitability of fruit seeds classified as biomass for the chemical reduction of metal oxide compounds will allow us to determine whether and to what extent they can be used as an alternative reductant to coke. This will enable us to determine how much the metallurgical process can be made to contribute to zero CO_2_ emissions.

Depending on the variety, biomass, including cherry stones, represents 8 to 15% of the total weight of the cherry fruit [5,6]. Between 2020 and 2023, the global production of cherries ranged from 3.8 to 4.36 million tons per year. This means that about 400,000 tons of waste cherry stones are generated annually. Biomass, including fruit stones, is mainly used for energy purposes, directly as fuel [7]. It can also be converted to gaseous and liquid biofuels [8,9]. Assuming that the same amount of CO_2_ is emitted during biomass combustion (including seeds) as plants absorb in photosynthesis, using seeds as fuel should be considered expedient. Also important is the fact that the cherry stones contain negligible amounts of sulfur (up to 0.1 wt. %) compared to traditional fuels, i.e., hard coal and coke (0.8 to 2.5 wt. %), which reduces the adverse phenomenon of sulfur oxide emissions to the environment. Analyzing the energy properties of cherry stones, it should be noted that their combustion heat has values similar to hardwood (23–27 MJ/kg) [10,11]. Additionally, their combustion produces negligible amounts of ash compared to other fuel materials (e.g., hard coal, lignite), which somewhat facilitates their combustion process in household heating stoves. At the same time, the ash can be used directly in horticulture.

Another application of cherry stones is in the production of activated carbon in the food industry [12,13]. The seeds are added in small quantities to feedstuffs, thus enriching dietary fiber and minerals [14,15]. They can also be used in cosmetics [16,17].

As part of the results presented in this article, an attempt was made to determine the possibility of replacing coke with a biomass raw material in the form of cherry seeds. So far, the literature lacks detailed information on the possibility of using this type of material in the pyrometallurgical process, where temperatures reach above 1000 °C. These conditions affect the formation of products at early stages, especially gaseous products, which can significantly impact the efficiency of the process and cause faster wear of the plant’s working components. This paper undertook a study of the characteristics of biomass in the form of cherry stones, which can affect the efficiency of the process. Slag from the process of obtaining copper, which, from the point of view of the closed-loop economy, is a valuable raw material in which the metals occur in oxide form, was used to verify the reduction capacity. The reductant’s behavior and the process’s efficiency allow us to conclude that it is a raw material with great technological potential. Still, its seasonal occurrence may carry certain limitations that require consideration of the preparation and logistics of obtaining and storing this type of material. The authors of the paper conducted several tests to assess the suitability of biomass raw materials, among which were the determination of energy parameters, thermogravimetric tests to determine the effect of temperature on the changes that occur, and tests mapping the actual reduction process. The article discusses the possibility of using biomass, such as cherry stones, as a reducing agent in recovering copper from metallurgical slags.

## 2. Materials and Methods

The research program that was conducted included a determination of the physicochemical properties, chemical composition, and the effect of high temperature on the reducing properties of the tested biomass. In this study, a three-stage research procedure was used to determine the suitability of biomass in the process of copper recovery from metallurgical slag using the reductive remelting method, through the following:

1. Determination of the combustion heat of cherry stones.
2. Thermogravimetric studies—determining the changes occurring in the biomass during heating in an inert and oxidizing atmosphere.
3. High-temperature reductive melting of metallurgical slags using biomass as a reductant in the form of cherry stones.

### 2.1. Research Materials

Biomass in the form of cherry stones was used as a reducing agent to perform experiments. The chemical composition is summarized in Table 1. The content of essential components was determined using the ELTRA CHS HELIOS analyzer (Eltra GmbH, Haan, Germany). The combustion temperature of the sample was 1400 °C. The results presented in Table 1 are the average values of five measurements of the test material. The cherry stones’ carbon, sulfur, and oxygen content were analyzed using the EDS energy dispersive X-ray spectrometry technique. For this purpose, the stones were ground, dried, and compressed into a pastil. The analysis was performed with a Thermo Noran EDS spectrometer (Inchinnan, UK), using a primary electron beam of 15 keV energy and 1.16 nA intensity. The study was conducted using standards from Astimex Standards Ltd., MINM25-53+FC (Toronto, Ontario, Canada), and correction of the results of quantitative analysis using the PROZA method (which involves fitting exponential curves describing the distribution of ionization as a function of distance from the material surface to the experimental results; this gives much better results for light elements and materials containing elements with significant differences in atomic numbers).

The central part of the reductive remelting study was carried out using industrial slag; the main components are shown in Table 2.

**Figure 1:**
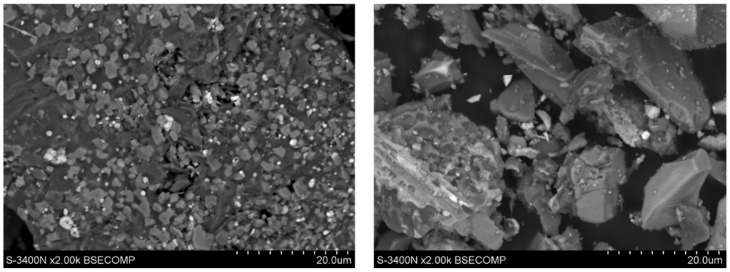
Morphology of the slag sample used in the tests.

The slag was analyzed to reveal the microstructure using a Hitachi S-3400N (Hitachi, Liverpool, UK) scanning electron microscope with an EDS Thermo Noran energy dispersive X-ray spectrometer. Imaged structures were taken with a BSE backscattered electron detector. Images of the slag structure are shown in Figure 1. From the images obtained, it was found that the slag was characterized by a powder-like structure with varying particle sizes in the range of about 1 µm–1 mm.

Additionally, to identify the phases present in the starting slag used in the study, it was subjected to phase analysis using an FPM XRD7 XRD (manufactured by Seifert-FPM, Rich. Seifert & Co. Freiberger Präzisionsmechanik GmbH & Co. KG, Freiberg, Germany) diffractometer. This analysis confirmed the presence of phases containing metallic components, copper, lead, and iron, which are characteristic of this type of slag.

Based on the phase analysis of the slag studied, the following phases containing Cu, Pb, and Fe were identified [18,19]:

- Maghemite Fe_1.966_O_2.963_ with a tetragonal structure;
- Cu_2_O cuprite with a regular structure;
- Al_2_PbSi_2_O_8_ with a single-stranded structure;
- PbO masicot with a rhombic structure.

**Figure 2:**
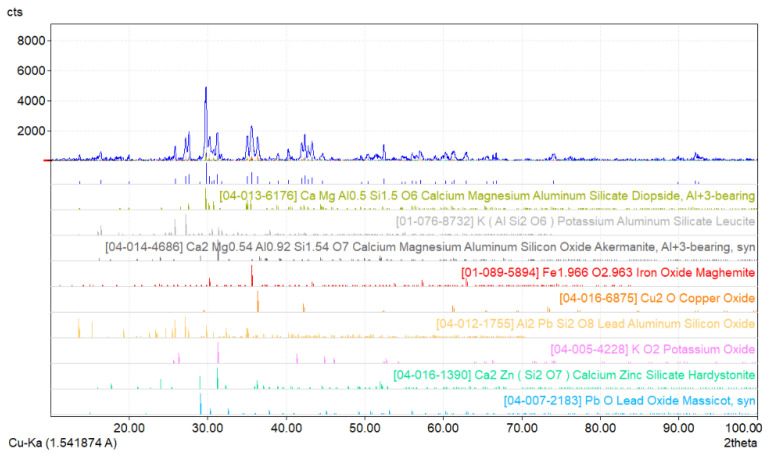
X-ray diffraction pattern of the initial slag sample.

Figure 2 shows the X-ray diffractogram of the sample of the studied slag.

### 2.2. Research Methodology

#### 2.2.1. Determination of Cherry Stones’ Combustion Heat

**Figure 3:**
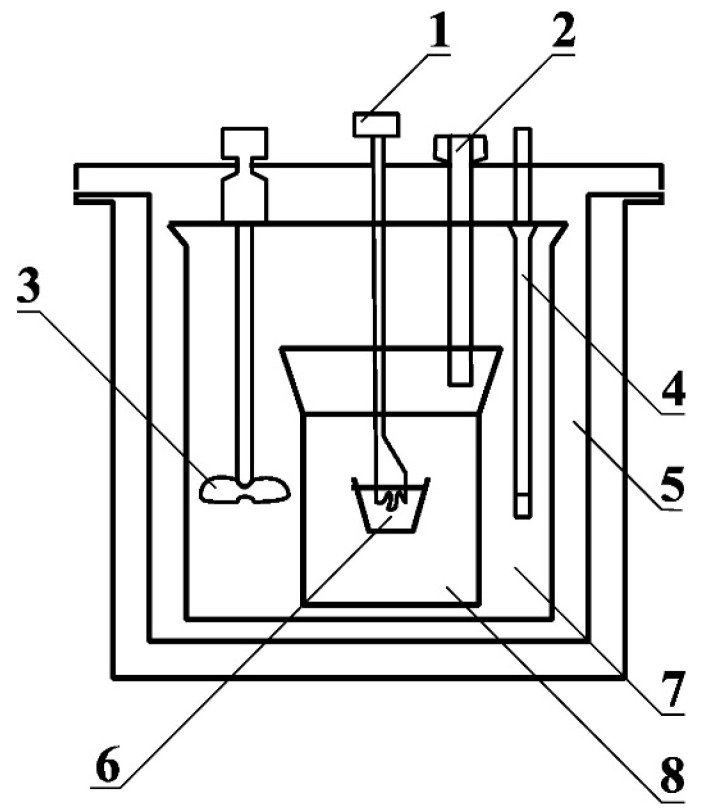
Caloric bomb scheme for combustion heat measurements according to EN ISO 1716 [20]: 1—energy source for firing wire, connected to ignition leads; 2—oxygen inlet; 3—stirrer; 4—temperature sensor; 5—outer (thermally insulated) water jacket; 6—crucible with test specimen; 7—calorimetric vessel with inner water jacket; 8—calorimetric bomb chamber.

One of the essential energy parameters characterizing fuels is the determination of the combustion heat. This is ‘the heat released with the maximum possible oxidation of a given fuel’, that is, with complete and total combustion. The determination of the combustion heat value was carried out according to EN ISO 1716 [20]. This test involves placing a powdered sample in a KL-12 Mn calorimetric bomb (Precyzja-BIT, Bydgoszcz, Poland—Figure 3) after weighing the sample’s weight and the incendiary wire’s weight. The working chamber is then filled with oxygen to create conditions for the complete and total combustion of the sample. The working chamber is placed in a double-water jacket so that it is possible to accurately determine the temperature changes that allow the determination of the combustion heat, including the heat coming from the condensation of water vapor produced during the combustion process. The ignition of the sample is triggered by an electrical system that allows an electric current to flow through the ignition wire. The data are recorded by a computer system, and particularly the water temperature in each jacket.

The value of the combustion heat is calculated from the following equation:

(1) $$PCS=\frac{E\cdot \left(T_{m}-T_{i}+C\right)-b_{i}}{m}$$

where

*PCS*—gross calorific value [MJ/kg];
*E*—water equivalent of the calorimeter, bomb, and its equipment, and water introduced into the bomb, read from the standard (PN-EN ISO 1716, 2010 [20]) [MJ/K];
*T*_m_—maximum temperature [°C];
*T*_i_—initial temperature [°C];
*C*—temperature correction taking into account exchange with the environment [°C]; in the method used, it was C = 0.0;
*b*_i_—correction for the combustion heat of the ignition wire [MJ], which is the product of the combustion heat of the wire b [MJ/kg] and m_d_ [kg];
*m*—mass of the test sample [kg].

#### 2.2.2. Thermogravimetric Analysis

Thermogravimetric testing is one method that allows the characterization of materials subjected to high temperatures. Capturing the changes associated with mass loss, determining the temperature ranges at which these changes occur, and analyzing the dynamics of mass changes make it possible to predict the behavior of a material under given conditions, depending on its intended use. Given that in pyrometallurgical processes, we are dealing with the impact of high temperature, it is necessary to characterize the material used so that it fulfills the intended role in the process. This is important because the gases emitted during the thermal decomposition of biomass can also be an essential factor that affects the efficiency of the processes carried out. The results obtained from the tests will make it possible to identify mass changes by mapping the aerobic and anaerobic conditions that may prevail in the working space of the reactor in which the process is carried out.

A TGA Q500 device (TA Instruments, New Castle, DE, USA) was used for thermogravimetric tests. It allows for testing of the course of the sample’s thermal decomposition in an oxidizing atmosphere and pyrolysis in an inert atmosphere. The device provides testing at sample heating rates from 0.01 to 100 K/min up to 1000 °C, with a claimed temperature measurement inaccuracy of 1 K at a measurement resolution of 0.1 K. The sample mass is measured at a resolution of 0.1 µg with a claimed measurement inaccuracy of 0.01%.

The test allowed the determination of characteristic temperatures: the onset of thermal decomposition/pyrolysis, the maximum rate of weight loss of the sample over time, and the mass of the residue after the test related to the content of noncombustible minerals. The tests were conducted in an air and high-purity nitrogen atmosphere. The sample heating rate was 100 K/min for a sample of 20–22 mg.

#### 2.2.3. Slag Reduction

Process efficiency studies using cherry stones were carried out by melting the slag reduction in an electric plunge resistance furnace type PT-40 300 (Czylok sp. z o.o, Jastrzebie-Zdroj, Poland), allowing the process to be carried out up to 1350 °C. This unit was adapted to load the crucible safely with the charge. The study’s variable parameter was the process’s duration (1–5 h). The duration of the process was selected based on the actual duration of the operations conducted under industrial conditions. In the study, 1300 °C was taken as the target temperature to carry out the experiments; this is the temperature used under the industrial conditions of copper smelters. Based on stoichiometric calculations, the amount of carbon needed to reduce the copper and lead oxides contained in the slag is about 3 g. The additional biomass weight of 18 g considered the excess carbon required for the Boudouard reaction to proceed, thus allowing for a reduction atmosphere in the device’s working chamber. A slag sample of 80 g was used in the experiments carried out.

At the end of the experiment, the weight of the smelted metal and the weight of the secondary slag were determined. Both the metal and the slag were subjected to chemical composition analysis to determine the content of the primary metals: copper, lead, and iron. The WDXRF technique with the SQX model-free analysis program was used to analyze the metal alloy components. The chemical composition of the slag was analyzed using an X-ray fluorescence spectrometer type Primus II (Rigaku, Neu-Isenburg, Germany). The measurement procedure was based on the PN-EN15309:2010 standard [21].

## 3. Test Results and Discussion

### 3.1. Combustion Heat

Table 3 summarizes the study results for the combustion heat of the cherry stones. It also summarizes the data available in the literature on the combustion heat values of stones from other fruits and coke, which is the primary fuel in pyrometallurgical processes.

The data presented in Table 3 show that the experimentally determined combustion heat value (*Q*_sp_—combustion heat, *Q*_sr_—average value) of the cherry stones ranged from 19535 to 20238 kJ/kg. Similar combustion heat values also characterize other types of biomass in the form of fruit seeds (apricot, peach, and plum). Furthermore, it was shown that the heat of coke combustion is significantly higher than that of the analyzed biomass. The difference in the values of the combustion heat of coke and biomass indicates that in the case of replacing the former with biomass in metallurgical processes, where it mainly plays the role of fuel, it will be necessary to supply a much more significant amount of biomass to obtain an equivalent amount of heat. This difference, however, is not so significant when coke or biomass directly or indirectly play the role of a reductant of metal compounds in the technological process.

Table 4 presents the chemical composition of cherry stone ash. As can be seen from the data presented, the main elements included in cherry stone ash are iron, potassium, calcium, and magnesium.

### 3.2. Thermogravimetric Analysis of Biomass

**Figure 4:**
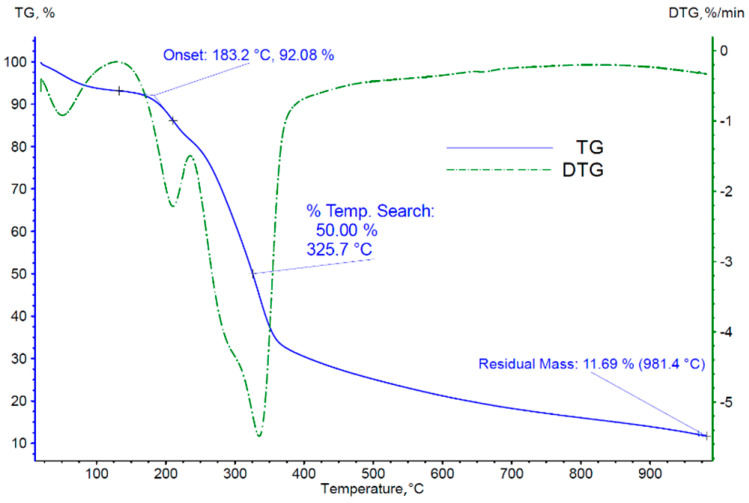
TG and DTG curves obtained from tests carried out in an inert atmosphere.

**Figure 5:**
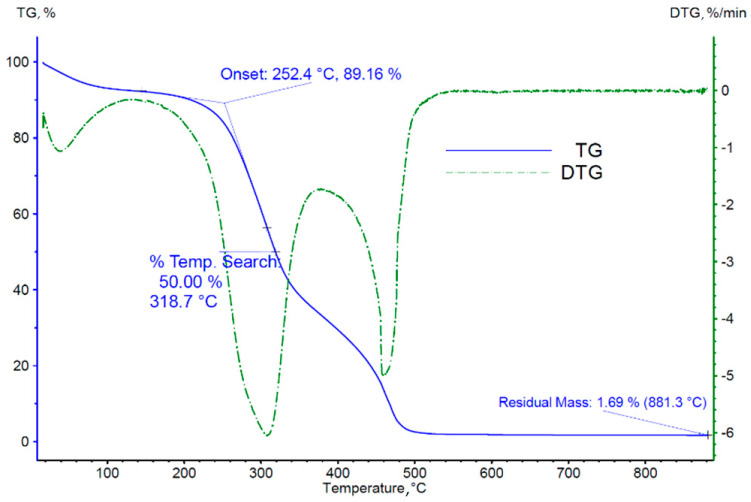
TG and DTG curves obtained from tests conducted in an oxidizing atmosphere.

Thermogravimetric results in the form of changes in the mass of the analyzed sample and the rate of these changes over time (TG and DTG curves) are shown in Figure 4 and Figure 5. Analyzing the TG and DTG curves obtained, the characteristic parameters of the process were determined:

(a) The temperature of onset of thermal decomposition;
(b) The temperature at which 50% of the sample mass is lost;
(c) The mass of the solid residue of the sample after reaching the set temperature;
(d) The temperatures of occurrence of individual peaks of the derivative of the sample mass loss over time, identified with the successive phases of thermal decomposition/pyrolysis of the tested material.

The results obtained in the form of TG and DTG curves for thermogravimetric studies of cherry stones for measurements conducted in both inert and oxidizing atmospheres are typical for materials of organic origin. At the first heating stage, we observed a significant mass loss, recorded in the temperature range from approximately 50 to 100 °C, resulting from the evaporation of transient moisture contained in the samples. The subsequent peaks of the DTG curve indicate the stages of pyrolysis (Figure 4) and thermal decomposition (Figure 5). Table 5 presents the appropriately estimated values of selected process parameters and the temperature ranges of the individual phases of thermal decomposition and pyrolysis.

Analyzing the TG and DTG curves obtained for cherry stones in experiments conducted in an inert atmosphere, it was found that three zones of mass loss characterize them and are typical for the degradation processes of various types of biomass [16]. The temperature change at the beginning of the decomposition of compounds present in the biomass significantly depends on its composition. The first slight mass loss (approximately 5% of mass) occurred at approximately 100 °C and was associated with the evaporation of transient moisture from the material. The mass loss (7.4%) up to 130 °C is related to the evaporation of the adsorbed moisture on the samples. However, such a mass loss is slightly higher than the moisture content of the initial samples (5.8 wt. %), suggesting that other biomass components are also removed from the biomass by extraction.

The second area, where the weight loss on the TG curve is most pronounced, characterizes the degradation of hemicellulose and cellulose. Analyzing the results obtained for an oxidizing atmosphere, the degradation area of the above biopolymers begins at 240 °C (513 K) and continues until 380 °C (653 K).

The third area features a slower mass loss, which can be attributed to the degradation of lignin in biomass. As seen during thermal degradation, the initial and final temperatures of the third zone were 380 °C (653 K) and 485 °C (758 K), respectively. In this area, degradation progresses with a gradually decreasing mass loss. At the end of the heating process, no more significant changes in the mass of the sample were observed. This is when only biocarbon and ash remain from the lignocellulosic structure.

For the measurement carried out in an inert atmosphere, the fundamental weight loss was approximately 60% of the sample weight, and the onset temperature was 269 °C. In comparison, the basic weight loss of the sample for a measurement in air was 70%, and the temperature at which the loss started was about 259 °C. The weight loss of the essential sample means the weight loss in the temperature range where the highest DTG value was recorded. This refers to the second stage of pyrolysis determined for the temperature range from 240 °C (513 K) to 380 °C (653 K).

Such a significant difference in the weight loss of the two samples is related to the different nature of the processes, and particularly the availability of oxygen in the sample’s surroundings. However, it should be noted that the DTG curve reached its extremes at 335 °C (608 K—inert atmosphere) and 310 °C (583 K—oxidizing atmosphere), which proves the reproducibility of the degradation process of cellulose and hemicellulose.

As shown in the study [17,25], of the gases formed in the pyrolysis of cherry stones in the gas phase, the main one was CO_2_ (up to 76–90% molar). The amount of methane was up to 15% molar. As the process temperature increased, the amount of CO and other hydrocarbon gases such as methane increased. The amount of CO_2_ decreased with increasing temperature, which was mainly due to the decomposition of cellulose and hemicellulose. CO is formed primarily through the secondary cracking of volatiles and the Boudouard reaction, such that a significant amount of CO_2_ and methane is produced. The gases produced during the pyrolysis process are very beneficial from the point of view of the cherry stones used in the slag reduction process. As noted above, the first of these gases is involved in the Boudouard reaction, which produces carbon monoxide, the primary reductant of the metal compounds in the slag. Methane is also a direct reductant of these compounds, and can contribute to the intensification of the reduction process itself. In addition, releasing significant amounts of these gases can cause the beneficial phenomenon of additional mixing of liquid slag, which promotes the separation of metal and slag droplets.

### 3.3. Copper Recovery Studies in the Process of Reductive Slag Remelting

During slag remelting with cherry stones as a reducing agent, we dealt with the reduction of metal oxides contained in the slag, i.e., mainly copper and lead oxides. The primary reducing agent in this case is carbon monoxide, formed in the process of decomposition of cellulose and hemicellulose, and as a result of the Boudouard reaction:

CO_2(g)_ + C = 2CO_(g)_ (2)

In addition, as described above, during the biomass decomposition process (cherry stones), methane CH_4_ can be formed, which can also be a reducing agent of copper, lead, and iron oxide compounds. To determine the feasibility of the reduction reaction of copper and lead oxides with methane during the slag melting tests discussed above, the values of the free enthalpy ΔG^0^_T_ and the change in enthalpy ΔH^0^_T_ for the following reactions were estimated:

4CuO + CH_4(g)_ = 4Cu + CO_2_(g) + 2H_2_O_(g)_ (3)

4Cu_2_O + CH_4(g)_ = 8Cu + CO_2(g)_ +2H_2_O_(g)_ (4)

4PbO + CH_4(g)_ = 4Pb + CO_2(g)_ + 2H_2_O_(g)_ (5)

4PbO + CH_4(g)_ = 4Pb_(l)_ + CO_2(g)_ + 2H_2_O_(g)_ (6)

6Fe_2_O_3_ + 5CH_4(g)_ =12Fe + 5CO_2(g)_ + 8H_2_O_(g)_ + 2H_2(g)_ (7)

4FeO + CH_4(g)_ = 4Fe + CO_2(g)_ + 2H_2_O_(g)_ (8)

Fe_3_O_4_ + CH_4(g)_ = 3Fe + CO_2(g)_ + 2H_2_O_(g)_ (9)

**Figure 6:**
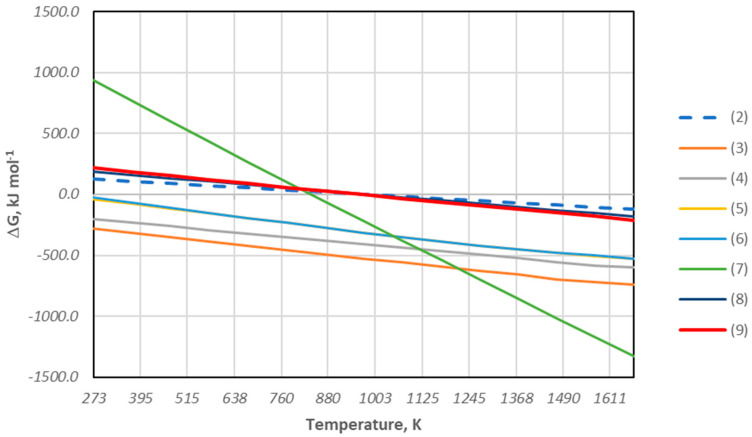
Change in the free enthalpy of reduction of metal oxides using methane as a reductant and the Boudouard reaction for reactions (2)–(9) from ambient temperature to 1400 °C.

**Figure 7:**
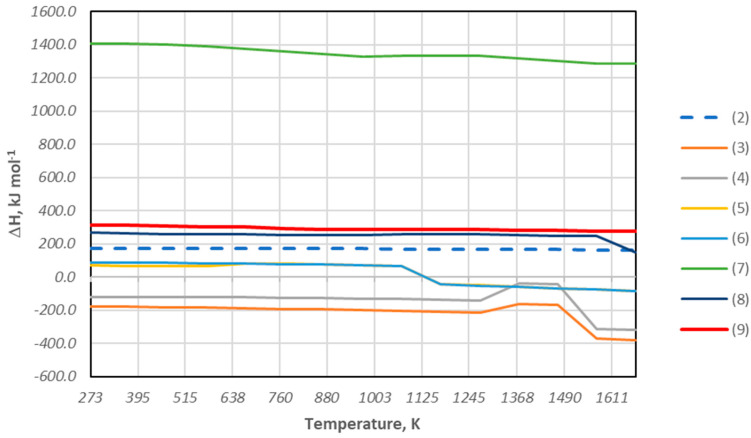
Change in the enthalpy of reduction of metal oxides using methane as a reductant and the Boudouard reaction for reactions (2)–(9) from ambient temperature to 1400 °C.

This was performed using the HSC Chemistry 6.1 program. Figure 6 and Figure 7 summarize the obtained values of ΔH^0^_T_ and ΔG^0^_T_ from room temperature to 1400 °C.

Based on the thermodynamic analysis, the data presented in the diagram (Figure 6) indicate that the determined values of the change in free enthalpy ΔG^0^_T_ for the reduction reaction of copper oxides and lead oxides with the use of methane, in the entire temperature range, take negative values, which confirms the possibility of the reduction reaction of these metals. On the other hand, the negative values of the free enthalpy ΔG^0^_T_ obtained for the reduction reaction of iron compounds are achieved above the temperature of 1073 K. This means that, from the thermodynamic point of view, the reduction of iron can occur above this temperature. Taking into account the course of the reaction and the exothermic or endothermic thermal effect, the magnitudes of the thermal effect in the form of enthalpy ΔH^0^_T_ accompanying the reaction of the course indicate that the energy demand necessary for the reduction reaction for both copper oxides and lead oxides is much lower than for iron oxides. This can be explained by the fact that copper and lead have a much lower affinity for oxygen than iron.

An indispensable tool for analyzing the efficiency of the slag reduction process studied was the determination of indicators that characterize the process. The most important of these are the degree of decopperization of the slag, the residue of lead and copper in the secondary slag, and the mass of the resulting alloy. In addition to the basic information on the amount and type of input materials in a given experiment, Table 6 shows the resulting data, i.e., the weights of the melted copper alloy and secondary slag. Table 7, on the other hand, summarizes the contents of copper, lead, and iron in the secondary slag, as well as the estimated values of the degree of decopperization of the slag, as well as the degree of transition of Cu, Pb, and Fe from the slag to the liquid alloy. The results of the analyses of the content of these metals in secondary slag were used for this purpose (Table 7). The value of the degree of slag decopperization was estimated from the equation

(10) $$U_{Cu}=\frac{m_{kCu}}{m_{0Cu}}\cdot 100\%$$

where

*m*_kCu_—weight of copper in secondary slag, [g];
*m*_0Cu_—mass of copper in slag undergoing a reduction process, [g].

The transition rate of Cu, Pb, and Fe from the slag to the alloy, on the other hand, was estimated from the equation

(11) $$S_{\left(Me\right)}=\frac{m_{0}\cdot C_{Me(0)}-m_{k}\cdot C_{Me(k)}}{m_{0}\cdot C_{Me(0)}}\cdot 100\%$$

where

*m*_0_, *m*_k_—initial and final mass of the slag after the process, respectively, g;
*C*_Me(0)_, *C*_Me(k)_—slag’s initial and final metal content, wt. %.

**Table 6:** Results of the slag reduction process.

Reducer	Time, h	Metal Mass, g	Secondary Slag Mass, g
Cherry stones	1	9.35	68.22
	1	9.74	66.37
	2	10.78	64.92
	2	10.18	63.74
	3	12.42	63.11
	3	12.59	62.66
	4	12.51	62.35
	4	12.44	62.88
	5	12.77	61.53

**Table 7:** Results of the copper slag reduction process using cherry stones.

Reducer	Time, h	Metal Content in Secondary Slag, wt. %			The Degree of Metal Transition from Slag to Alloy, %			Degree of Decopperization, %
		Cu	Pb	Fe	Cu	Pb	Fe	
Cherry stones	1	1.03	2.05	10.96	90.25	24.41	18.15	90.00
	1	1.33	1.61	10.72	88.98	38.98	17.71	87.08
	2	0.25	1.04	10.48	98.03	62.49	25.64	97.57
	2	0.42	0.89	10.99	96.75	68.48	21.18	95.92
	3	0.24	0.94	10.18	98.17	67.27	28.23	97.66
	3	0.28	0.43	10.29	97.85	84.93	26.93	97.28
	4	0.18	0.72	10.53	98.63	75.06	26.13	98.25
	4	0.25	0.39	10.89	98.09	86.37	22.95	97.57
	5	0.21	0.43	9.98	98.13	85.19	25.91	98.43

**Figure 8:**
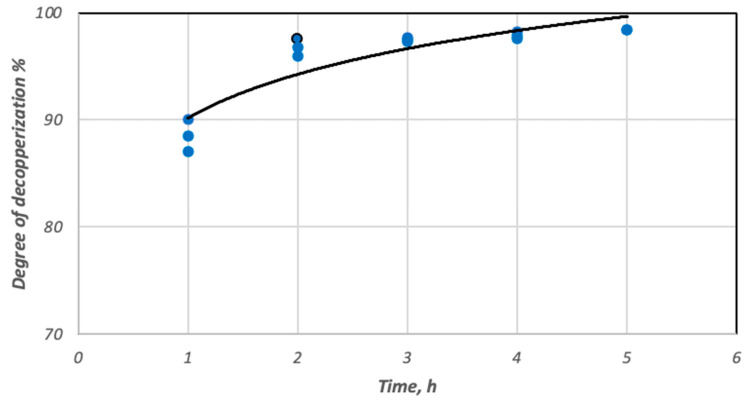
Change in the degree of slag decopperization depends on the duration of the reduction process.

Figure 8 shows a graphical interpretation of the change in the degree of slag decopperization as a function of the duration of the reduction process.

**Figure 9:**
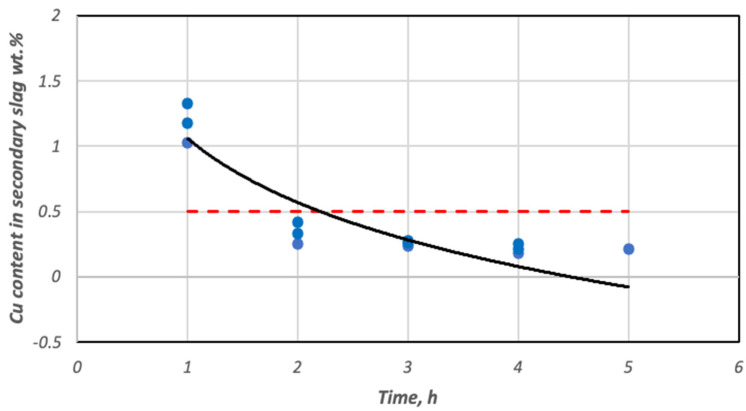
Change in copper content in secondary slag depending on the duration of the reduction process.

In the method adopted for estimating the degree of decopperization of slag with a high content of this metal in the starting slag, the values of this parameter can be obtained even above 95% for significant values of copper in the secondary slag (slag after reduction). For this reason, it became necessary to supplement the analysis of the reduction melt results with the determination of the effect of the process reduction duration on the copper content in the secondary slag. Figure 9 shows a graphical interpretation of this relationship. In addition, the figure also shows an additional dashed line showing the maximum copper content below which the slag obtained from the de-greasing process can be considered waste.

**Figure 10:**
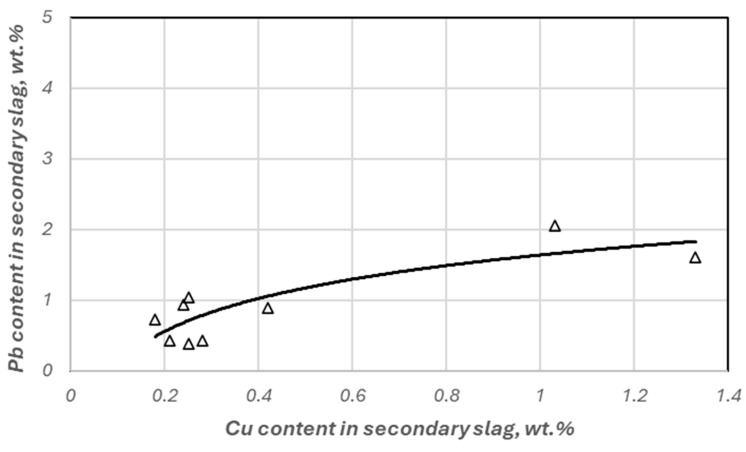
Change in lead and copper content in secondary slag.

Figure 10 shows the correlation, determined from experimental data, between the copper content of the secondary slag and the lead content of the slag. It shows that the reduction of lead oxide compounds intensifies when the copper content of the slag drops below 0.4 wt. %. Thus, the pronounced selectivity of the reduction of lead oxides to Cu_2_O results in a low concentration of this metal in the melt.

**Figure 11:**
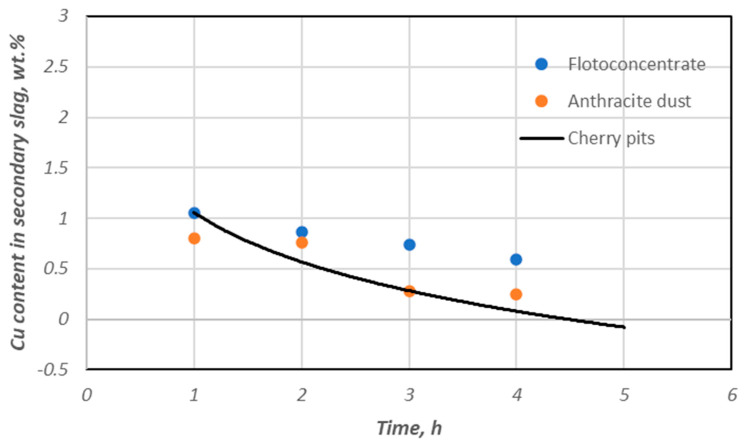
Change in the copper content in secondary slag depends on the duration of the reduction process, considering the share of various carbon reducers.

The slag reduction results obtained with cherry stone biomass are similar to those obtained with fine coal-bearing materials (anthracite dust, flotoconcentrate). The data summarized in Figure 11 illustrate this.

These data show that the proposed type of biomass can be a substitute for traditional coal-bearing materials in the process discussed.

The estimated values of the degree of transition of metals to the alloy after the slag reduction process ranged from 88 to 98% for copper, 24 to 86% for lead, and 17 to 28 for iron (Table 6). Considering the thermodynamic description of the obtained values of free enthalpy, the reaction will be ordered according to the lowest values of this parameter. It is clear that as the content of a given element decreases, its activity decreases. Thus, the magnitudes of these parameters change, affecting favorable conditions for the remaining reactions. It is worth noting that the determined heat effect in the form of enthalpy for the analyzed reactions allows us to identify the thermal demand, that is, the energy necessary for the occurrence of the analyzed reduction process for individual elements. Again, for the analyzed system, the highest energy requirement is shown by iron, and the lowest on the order of −200 kJ/mol for copper oxides.

**Figure 12:**
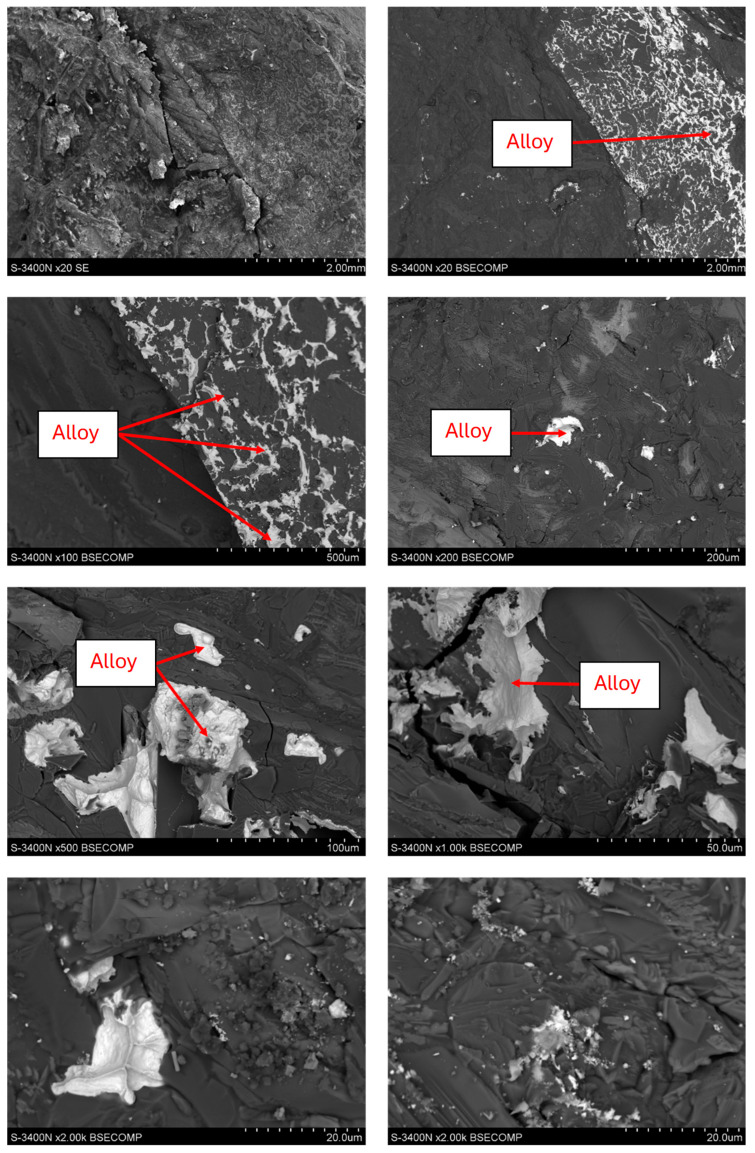
Morphology of the secondary slag sample after the reduction process.

As noted earlier, the secondary slags were also subjected to microstructure analysis, which was performed using a scanning electron microscope. These showed the presence of metallic alloy precipitates in them (Figure 12). The process results obtained in the form of both copper and lead content in the secondary slag allow these materials to be used in other industries or safely deposited. The remaining metal trapped in the slag results from significant fragmentation and thus suspension in the slag volume. To improve the separation of metal from the slag, it would be necessary to increase the sedimentation time of the material at a high temperature or to induce stirring of the bath to unite the metal droplets and more easily separate them. Analyzing the results obtained for reducing metal-bearing material from the production process indicates that using a coke substitute can point to the potential and benefits of using biomass. The first advantage of such a solution is the reduction in the exploitation of non-renewable deposits and the reduction in the carbon footprint associated with balancing the amount of CO_2_ emitted with that absorbed by growing plants. Another positive effect is the link between the agricultural industry and the industrial one, which has excellent potential for the use and processing of industrial waste. However, this type of solution may have some risks related to the seasonality of agricultural production and the strong dependence of agricultural production volumes on weather conditions. In this case, solutions should be sought to secure the supply or expand the spectrum of biomass raw materials that can be used equally seriously in metal production processes.

## 4. Conclusions

This article presents the results of a study on the copper slag decopperization process using cherry stones as a biomass reducer. The results showed the possibility of replacing fine-grained carbon reducers used in metal recovery from metallurgical slags (coke and anthracite dust). The degree of decopperization of the slag studied reached values of 87 to 98% and 85.3% for Cu and Pb. The content of copper and lead in the secondary slag after reduction dropped from 10.3 wt. % to 0.18 wt. % and from 2.25 wt. % to 0.39 wt. %, respectively.

Estimated values for the degree of transition of metals to alloy after the slag reduction process ranged from 88 to 98% for copper, 24 to 86% for lead, and 17 to 28 for iron, respectively.

In the study, it was assumed that the prerequisite for completing the reduction process was that the copper content of the secondary slag reached below 0.5 wt. %. In the case of the realized tests, this level was achieved practically after just 2 h of the reduction process. Further extension of the reduction process beyond 4 h was considered inefficient. As a result of its elevated lead content, the obtained metallic alloy should be directed to the conversion process. Secondary slag containing less than 0.5 wt. % copper and lead meet environmental standards and can be waste slag used in road construction, among other applications.

It should be noted that, unlike conventional fossil fuels, the use of cherry stones to reduce copper slag can be considered an environmentally neutral method of carbon offset. The resulting secondary slag is a waste product that can be stored and disposed of without harmful environmental effects due to its low lead content.

The problems that need to be solved are the form of biomass and the possible significant loss of biomass in the relatively early stage of heating feedstock in the industrial process. This requires large-scale laboratory or semi-technical scale studies.

## Figures and Tables

**Table 1:** Chemical composition of biomass used in research.

Material	Element Content, % by Mass					
	C	S	Cl	N	H	O_2_
Cherry stones	49.11–53.8	0.02–0.08	-	4.34	7.05	37.6–39.1

**Table 2:** The main components of industrial slag used in the research.

**Slag Component**	Cu	Pb	Fe	SiO_2_	CaO
**Component Content, % by Weight**	10.30	2.25	11.11	34.50	14.12

**Table 3:** Combustion heat of fruit stone biomass.

Sample	*Q_sp_*, kJ/kg	*Q_sr_*, kJ/kg	Comments
Cherry stones	19,535	19,995	Own research
	20,211		
	20,238		
Cherry stones	-	19,540–22,020	[5,7,22]
Apricot kernels	20,390–20,656	-	[23]
Peach stones	20,820–22,082	-	[23,24]
Plum stones	20,760–22,460	-	[11]

**Table 4:** Chemical composition of cherry stone ash.

Material	Element Content, % by Mass					
	Fe	K	Ca	Mg	Cu	Na
Cherry stones ash	15.65	13.01	12.68	4.98	0.62	0.49

**Table 5:** Recorded mass losses of the particular samples.

Sample Mass Loss	Nitrogen			Air		
	Loss I, %	Loss II, %	Loss III, %	Loss I, %	Loss II, %	Loss III, %
	8	13	47	5	55	15
The temperature range for the particular loss, °C	≤130	130–240	240–400	≤150	240–380	380–485
Maximum mass loss rate, %/min	0.95	2.21	5.52	1.82	6.04	5.01

## Data Availability

The original contributions presented in this study are included in the article. Further inquiries can be directed to the corresponding author.
